# Successful extended use of Impella 5.5 as a bridge to heart transplantation

**DOI:** 10.1093/jscr/rjad262

**Published:** 2023-06-05

**Authors:** Omar M Sharaf, Hannia P Diaz-Ayllon, Elisha M Myers, Mustafa M Ahmed, Mark S Bleiweis, Eric I Jeng

**Affiliations:** Division of Cardiovascular Surgery, Department of Surgery, University of Florida Health, Gainesville, FL, USA; College of Medicine, Florida Atlantic University, Boca Raton, FL, USA; College of Medicine, Florida Atlantic University, Boca Raton, FL, USA; Division of Cardiovascular Medicine, Department of Medicine, University of Florida Health, Gainesville, FL, USA; Division of Cardiovascular Surgery, Department of Surgery, University of Florida Health, Gainesville, FL, USA; Division of Cardiovascular Surgery, Department of Surgery, University of Florida Health, Gainesville, FL, USA

## Abstract

We present the case of a 60-year-old gentleman who was admitted with acute-on-chronic cardiogenic shock and was supported with axillary Impella 5.5® for 123 days prior to heart transplantation. Total length of temporary mechanical circulatory support (MCS) was 132 days, which included 9 days with an intra-aortic balloon pump prior to Impella. During support, the patient remained extubated, participated in regular ambulation and rehabilitation with physical therapy and had continuous monitoring of device positioning. He did not experience any vascular or septic events while on temporary MCS and had improved hemodynamics and renal function after Impella initiation. Post-transplantation course was uncomplicated, and he is doing well without evidence for allograft dysfunction over 581 days post-transplantation. To our knowledge, this is the longest Impella 5.5®-supported patient during the new United Network for Organ Sharing Heart Allocation era who was successfully bridged to heart transplantation with over 1-year follow-up.

## INTRODUCTION

Patients with refractory cardiogenic shock may be stabilized with temporary mechanical circulatory support (MCS) as a bridge to recovery, long-term durable MCS and/or heart transplantation. Various forms of temporary MCS exist, including but not limited to intra-aortic balloon pump (IABP), Impella, CentriMag™, LifeSPARC® and extracorporeal membrane oxygenation. These devices are each associated with unique risks and are FDA-approved for varying support durations. The United Network for Organ Sharing (UNOS) reorganized the Heart Allocation guidelines in 2018, resulting in a shift in bridging strategies centers employ to optimize patient outcomes.

Impella 5.5® is implanted using an axillary or direct aortic approach via sternotomy or thoracotomy. It was designed to provide short-term MCS and is currently approved by the FDA to be used for ≤14 days [1]. However, many centers have reported successful bridge beyond this duration [2–7]. We report a patient with cardiogenic shock supported with Impella 5.5® for the longest known duration in the United States as a successful bridge to heart transplantation.

## CASE REPORT

A 60-year-old gentleman with nonischemic cardiomyopathy, atrial fibrillation, diabetes mellitus and hypertension was admitted with NYHA Class IV symptoms. Right heart catheterization and laboratory findings revealed elevated biventricular pressures, depressed cardiac index (CI) and signs of poor end-organ perfusion, consistent with acute-on-chronic cardiogenic shock. Dobutamine was started. On inotropes, pulmonary artery pressure (PAP) was 57/34 mmHg, pulmonary capillary wedge pressure (PCWP) was 26 mmHg and CI was 1.4 L/min/m [2]. Laboratory findings revealed elevated creatinine (2.21 mg/dl; baseline of <1 mg/dl), lactate (2.6 mmol/L), aspartate transaminase (138 IU/L) and alanine transminase (170 IU/L).

**Figure 1:**
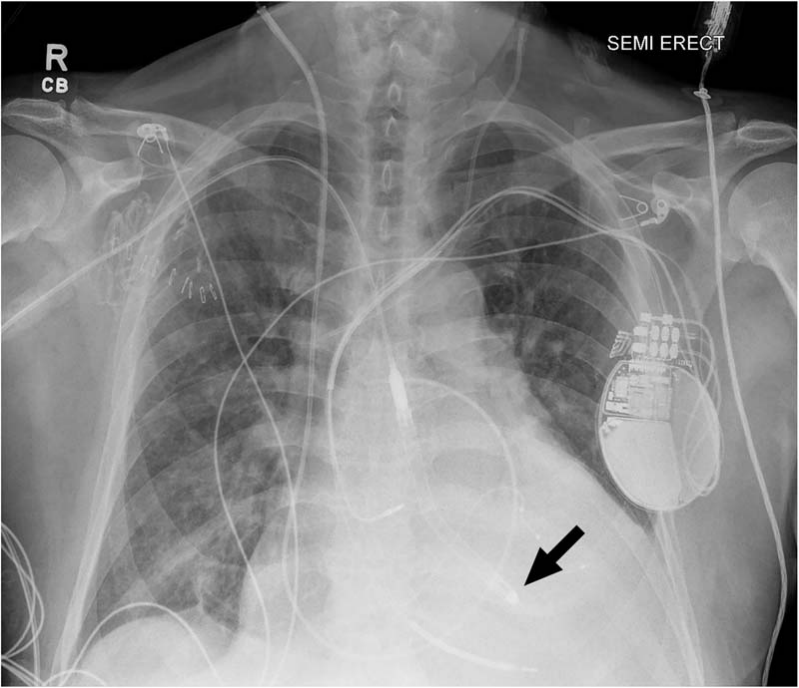
Chest radiograph after Impella 5.5® placement demonstrating adequate placement across the aortic valve into the left ventricle. The arrow indicates the tip of the Impella positioned in the left ventricle.

Despite escalating inotropic support and diuresis, the patient deteriorated—a femoral IABP was inserted on hospital day 3. Despite support with IABP, he remained with evidence of end-organ dysfunction and refractory cardiogenic shock. After 9 days of support, his MCS platform was upgraded to an axillary Impella 5.5® to provide better systemic support with active unloading of the left ventricle (Fig. 1). Hemodynamic parameters improved (PAP, 35/15 mmHg; PCWP, 13 mmHg; CI, 3.5 L/min/m [2]), and creatinine decreased to baseline. The Medical Review Board consensus was bridge to heart transplantation as UNOS Status 2.

**Figure 2:**
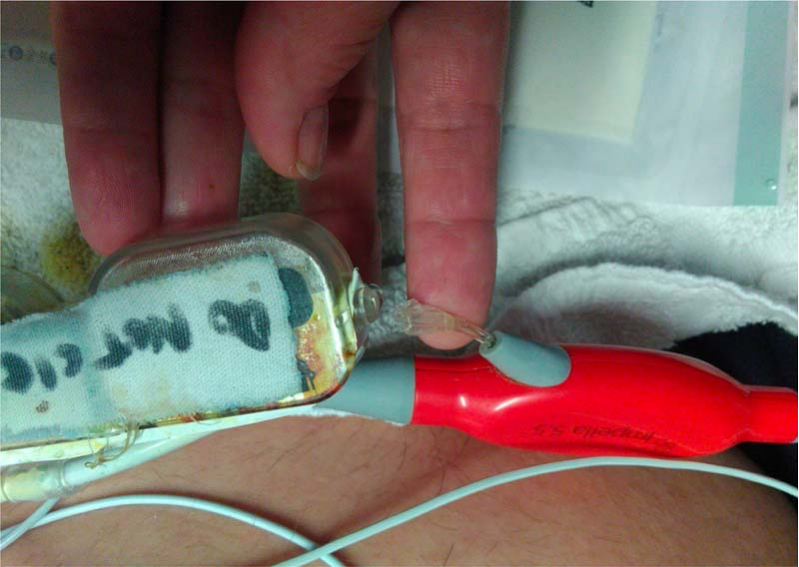
Break in the Impella 5.5 sidearm line from the reservoir to the device.

**Figure 3:**
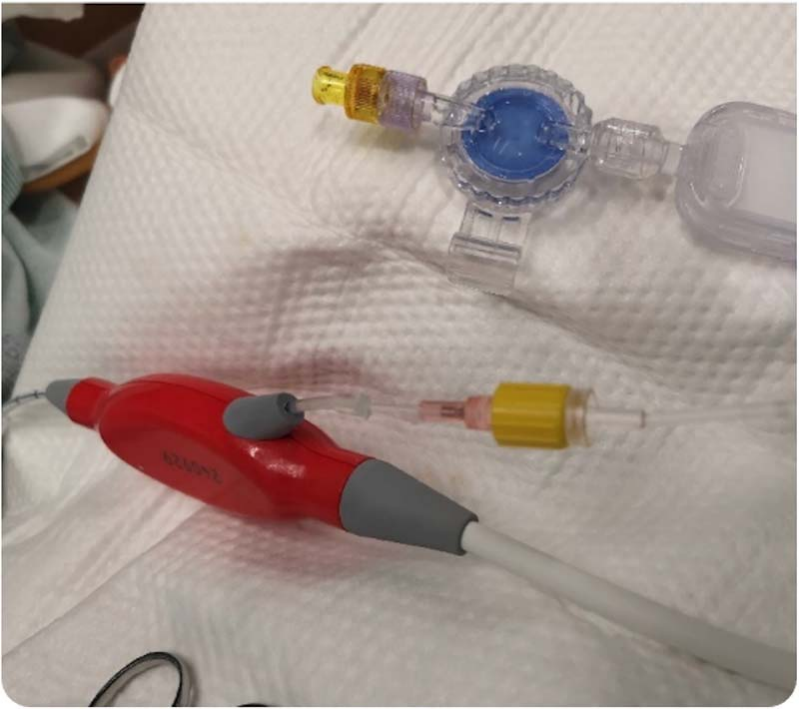
Removal of the purge reservoir and reestablishment of the purge line using a 20-gauge intravenous starter needle.

**Figure 4:**
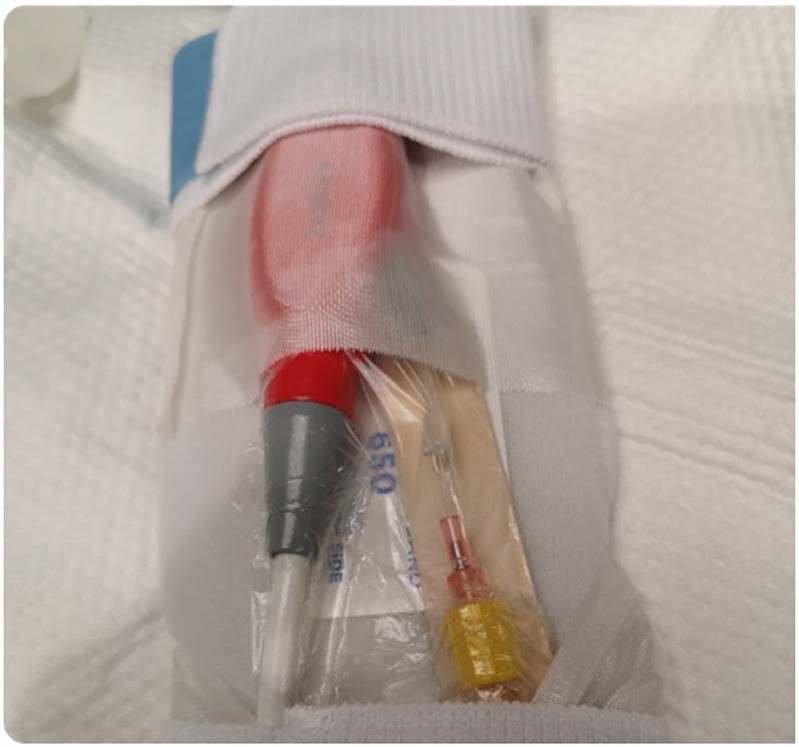
Securing the Impella system after purge sidearm modification.

During support, the patient was ambulatory and remained extubated. Daily radiographs ensured appropriate device positioning. The patient did not experience device-related complications while supported; however, after being supported for 114 days, the Impella purge sidearm line from the reservoir to the++ pump broke (Fig. 2), alarming the system. This required removal of the purge reservoir, which functions to provide 90 s of continuous fluid purge during purge fluid bag exchange. Modification of the purge line was then performed to allow purge fluid to continue to cool the device motor—this was achieved using a 20-gauge IV starter needle to thread an IV into the remaining tubing, which was then connected to the purge fluid line (Fig. 3). This system was then secured using a tongue depressor, arm board, and adhesive tape and Velcro (Fig. 4). Without a purge reservoir, continuous flow was achieved with fluid bolus prior to bag exchange.

After 123 days of Impella 5.5® support, the patient underwent orthotopic heart transplantation with Impella explant. The post-transplantation hospital course was uncomplicated, and he was discharged home on postoperative day 20. Endomyocardial biopsies were performed at three weeks, two months and three months after the transplant, and showed no signs of rejection. (ISHLT Grade 0R). The patient was last seen over 581 days postoperatively, doing well with preserved allograft function.

## DISCUSSION

Impella 5.5® has several improvements from predecessors, including increased rigidity, shorter length and lack of a pigtail catheter on the ventricular end [5, 8]. These improvements address its deliverability and durability [8]; yet, Impella 5.5® is FDA-approved for ≤14 days. Recent evidence suggests this device may be used to successfully bridge patients beyond the approved duration [2, 3, 5–8].

Most series evaluating prolonged Impella 5.5® use report support durations of <90 days, although one multicenter study in Germany reported a support duration of 164 days in one patient [8]. Ramzy *et al*. evaluated 200 patients supported across 42 US centers [7]. In that study, the median support duration was 10 days, maximum support duration was 64.4 days and overall survival was 74%. However, there were no analyses comparing outcomes based on support duration. In another study of Impella 5.5®, 24 patients were supported for 26 episodes of support [6]. Of these patients, 10 patients were supported for >14 days for 11 episodes of support with a maximum support duration of 80 days. The overall complication rate for those on prolonged support was 73%, consisting of axillary hematoma, device malfunction and gastrointestinal bleed. Additionally, the survival rate for Impella 5.5® when used for >14 days was 67%. These findings suggest that prolonged Impella 5.5® use may be associated with a significant risk of complications and mortality. To our knowledge, the longest duration of Impella support that has been reported in the literature was 164 days in Germany [8]; however, that patient lacked long-term follow-up. In that study, Bernhardt *et al*. evaluated the early outcomes and adverse event profile of Impella 5.5® among 46 patients across multiple centers in Germany. The patient supported for 164 days had an underlying diagnosis of hypertrophic cardiomyopathy and underwent successful transplantation. Various independent series documenting effective Impella 5.5® use beyond FDA-approved guidelines suggests there may be center-specific strategies associated with successful bridging. Our case of extended duration Impella 5.5® support (123 days) as successful bridge to heart transplantation highlights the potential of this technology. We believe that encouraging early ambulation, employing a regimen of physical therapy to prevent deconditioning while on temporary MCS, limiting duration of mechanical ventilation and vigilant monitoring of device position contributed to successful bridging.

Our report highlights one of the longest use durations of an Impella 5.5® and illustrates that extended use beyond the FDA-approved 14 days is achievable with excellent mid-term outcomes. Regular ambulation, limiting mechanical ventilation and continuous monitoring of device positioning were key. Although this singular case had a favorable course, larger multicenter studies are needed to evaluate the efficacy, morbidity and mortality associated with extended Impella 5.5® for bridge to transplantation.
